# The prognostic effect of ST-elevation in lead aVR on coronary artery disease, and outcome in acute coronary syndrome patients: a systematic review and meta-analysis

**DOI:** 10.1186/s40001-022-00931-5

**Published:** 2022-12-21

**Authors:** Erfan Kazemi, Ali Mansoursamaei, Morteza Bijan, Ali Hosseinzadeh, Hossein Sheibani

**Affiliations:** 1 Student Research Committee, School of Medicine, Shahroud University of Medical Sciences, Shahroud, Iran; 2 Department of Epidemiology, School of Public Health, Shahroud University of Medical Sciences, Shahroud, Iran; 3 Clinical Research Development Unit, Imam Hossein Hospital, Shahroud University of Medical Sciences, Imam Ave., Shahroud, 3616911151 Iran

**Keywords:** ST-elevation, aVR lead, Death, Coronary artery disease

## Abstract

**Background:**

Rapid diagnosis of coronary artery disease has an important role in saving patients. The aim of this study is to evaluate if aVR lead ST-elevation (STE) can predict LM/3VD, left main (LM) disease, and three-vessel disease (3VD), outcome in acute coronary syndrome (ACS) patients.

**Methods:**

In this systematic review and meta-analysis, 45 qualified studies were entered. Scopus, Pub med, Google scholar, Web of science, Cochrane library were searched on 12 November 2021.

**Results:**

This systematic review includes 52,175 participants. In patients with STE, the total odds ratios for LM, 3VD, and LM/3VD were 5.48 (95% CI 3.88, 7.76), 2.21 (95% CI 1.78, 3.27), and 6.21 (95% CI 3.49, 11,6), respectively. STE in lead aVR was linked with in-hospital death (OR = 2.99, CI 1.90, 4.72) and 90-day mortality (OR = 3.09, CI 2.17, 4.39), despite the fact that it could not predict 30-day mortality (OR = 1.11, CI 0.95, 1.31). The STE > 1 mm subgroup had the highest sensitivity for LM (0.9, 95% CI 0.82, 0.98), whereas the STE > 0.5 mm (0.76, 95% CI 0.61, 0.90) subgroup had the highest sensitivity for LM/3VD. The appropriate cut-off point with highest specificity for LM/3VD and LM was STE > 1.5 mm (0.80, 95% CI 0.75, 0.85) and STE > 0.5 mm, respectively (0.75, 95% CI 0.67, 0.84, *I*^2^ = 97%).

**Conclusion:**

The odds of LM and LM/3VD were higher than 3VD in ACS patients with STE in lead aVR. Also, STE > 0.5 mm was the best cut-off point to screen LM/3VD, whereas for LM diagnosis, STE > 1 mm had the highest sensitivity. Furthermore, LM/3VD had a higher overall specificity than LM.

**Supplementary Information:**

The online version contains supplementary material available at 10.1186/s40001-022-00931-5.

## Introduction

One of the main reasons of death in the worldwide adult population is ischemic heart disease (IHD) that imposes a significant financial burden on the health care system [1, 2]. Almost, 40% of patients with IHD present with acute coronary syndrome (ACS), which includes acute non-ST elevation myocardial infarction (NSTEMI), unstable angina, and ST-elevation myocardial infarction (STEMI). ACS will rise exponentially in the coming years as result of the rising prevalence of some risk factors such as diabetes, obesity as well as increasing the life expectancy of the worldwide population [3]. A significant proportion of ACS patients have left anterior coronary artery (LAD), left main coronary artery stenosis (LMCA), or both of them [4]. Obstruction in these arteries critically decrease coronary flow, which impair left ventricular function, leading to adverse outcomes and intraoperative complications, so early detection of these lesions is critical. Patients with ACS (LM/3VD) are at high risk for short-term and long-term cardiovascular side effects [5]. Despite remarkable progress in medical diagnosis, the electrocardiogram (ECG) is still the primary diagnostic tool in ACS patients. Valuable information is accrued of ECG in order to early detection of damaged coronary artery area, myocardial ischemia, lesion identification, and extent of infarction. Additionally, ECG can help predict possible complications [6].

Lead aVR has been long forgotten until recent years unlike the other 11 leads. Although recent examinations have stated that ST-segment change analysis in lead aVR provides helpful data on the coronary angiographic anatomy and risk classification in ACS [7]. Previous studies have shown that an increase in the ST-segment in the aVR lead (STE-aVR) might be helpful in order to diagnose left main disease or three-vessel disease (3VD) [4, 5, 8], despite some limitations including the selection bias, the retrospective nature of the studies, and the small sample size. Therefore, the aVR lead changes are not included in the American Heart Association's latest scientific statement on ECG interpretation [10]. The aim of this systematic review and meta-analysis was to investigate the diagnostic role of STE-aVR in ACS patients and its association with LM disease and 3VD.

## Methods

### Search strategy

Scopus, Cochrane library, Pub Med, Google scholar, Web of science, were searched on 12 November 2021 with following strategy: St-elevation[Title/Abstract] AND (Angiography[Title/Abstract] OR “Angiography result”[Title/Abstract] OR “Left main disease” OR “3VD” OR “3 vessel disease” OR “Myocardial infarction” OR “Left main”[Title/Abstract]) AND (“aVR lead”[Title/Abstract] OR aVR[Title/Abstract]).

### Selection process and eligibility criteria

All articles were divided into three groups. Then, three authors (by E.K, A.M, and M.B) screened the article base on title, abstract, and keywords independently. Studies fulfilling the entire inclusion criteria entered in the study. Besides, there was no limitation in terms of language of article. Eligible criteria: (1) cohort, cross-sectional, and case–control studies were enrolled; (2) the enrolled studies were adopted from articles with acute coronary syndrome (ACS) study population; (3) studies reported odds ratio (OR) or sensitivity /specificity to predict LM or 3VD or LM/3VD or death base on the size of aVR ST-elevation. Some studies did not report any OR, although they had essential data for calculation of OR. Consequently, they were included in the study.

### Extraction process and quality assessment

E.K, A.M, and M.B. did extraction process and quality assessment of article independently. Checklist used to assess the quality of studies was Appendix 2: MINORS Criteria. Non-comparative studies and comparative studies have 8 and 12 criteria, respectively. The items were scored in this way: (1) not reported = 0, (2) report but inadequate = 1, (3) completely reported = 2. The total ideal score was 16 for non-comparative studies and 24 for comparative studies [10].

### Analysis

OR was used as a common correlation index to assess the strength of the relationship. Forest plots were drawn to examine the modified ORs along with their confidence intervals. The meta-analyses were performed using the fixed-effects or random-effects method to estimate the summary OR and 95% of CI using the inverse-variance weights and the DerSimonian–Laird estimator, respectively. The heterogeneity was evaluated by Q-Cochran test at the significance level of 0.05 and index *I*^2^ among studies. For *I*^2^ ≥ 50% and *P* ≤ 0.05, heterogeneity was considered statistically significant. Meta-regression and subgroup analysis were performed to identify the source of heterogeneity. Subgroup analysis was done based on different sizes of STE in lead aVR and time of death. Publication bias was assessed the publication bias. In the funnel plot, ORs were plotted against the inverse of the square of the standard error. All analyses were done using STATA 14.0 software. All *P* values were two-tailed. Also, the significant level of *p* value was less than 0.05.

## Result

### Study selection and characteristics

**Fig. 1:**
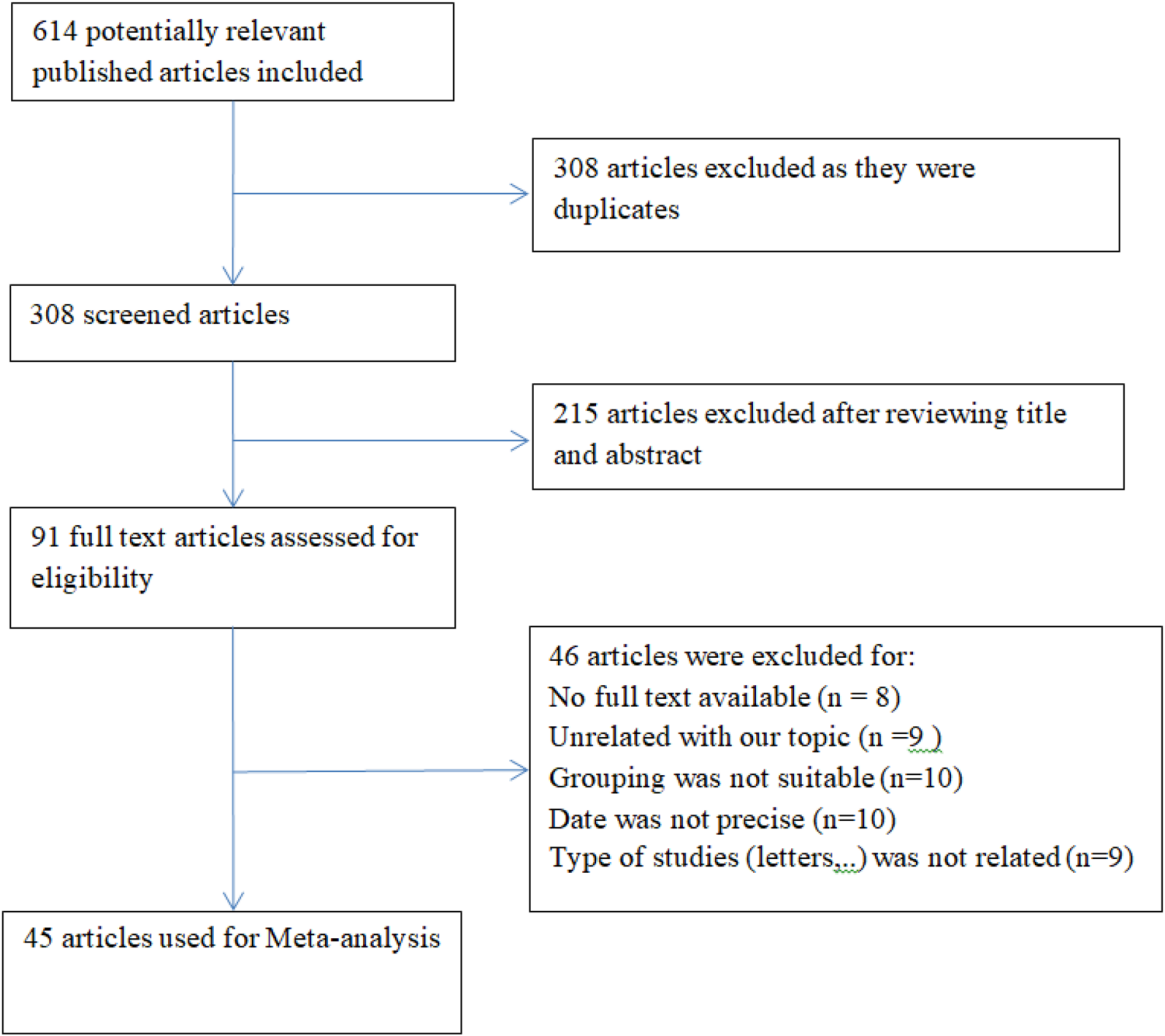
The diagram shows research process

Six-hundred fourteen related studies were extracted initially. Duplicate articles (*n* = 318) and studies that could not fulfill the study inclusion criteria (*n* = 264) were excluded after title, abstract and full-text screening (Fig. 1). Finally, 45 qualified articles were entered in this study [5, 11–54]. The total participants of the included studies were 52,175. All of the eligible studies were performed on both men and women. Table 1 summarizes the characteristics and scores of eligible researches.

**Table 1:** The descriptive data of eligible studies. The unit of ST-elevation size is millimeter

Author and publish year	Duration of study (year)	Country	Type of study	Number of patients and grouping	Total mean age ± SD	Male/female	Score of quality assessment
Ashraf. H et al. (2011) [29]	1	Egypt	Cross-sectional	81 ST-elevation AVR ≥ 0.5 mm 69 non-ST-elevation AVR	59 ± 9	115/35	16
Rathi. N et al. (2016) [41]	1	Pakistan	Cross-sectional	81 ST-elevation AVR ≥ 0.5 mm 33 non-ST-elevation AVR	56.65 ± 15.44	88/26	14
Katırcıbaşı. T. M et al. (2007) [30]	6	Turkey	Cross-sectional	65 ST-elevation AVR ≥ 1 mm 39 non-ST-elevation AVR	60.6	79/25	14
Kosuge. M et al. (2008) [33]	1	Japan	Cross-sectional	92 ST-elevation AVR ≥ 0.5 mm 275 non-ST-elevation AVR	67 ± 10	252/115	15
Rostoff. P et al. (2006) [42]	Unclear	Poland	Cross-sectional	54 ST-elevation AVR ≥ 0.5 mm 80 non-ST-elevation AVR	59.9 ± 9.7	92/42	12
Nabati. M et al. (2017) [38]	1	Iran	Cohort	52 ST-elevation AVR ≥ 0.5 mm 77 non-ST-elevation AVR	58.40 ± 10.64	65/64	13
Y. M. Marrero (2020) [37]	3	Cuba	Cross-sectional	34st-elevation AVR ≥ 0.5 mm 78 non-ST-elevation AVR	62.5 ± 12.4	68/44	13
Kosuge. M et al. (2009) [32]	Unclear	Japan	Cross-sectional	345 ST-elevation AVR ≥ 0.5 mm 156 non-ST-elevation AVR	66 ± 11	348/153	14
Kosuge. M et al. (2005) [35]	4	Japan	Cross-sectional	83 ST-elevation AVR ≥ 0.5 mm 227 non-ST-elevation AVR	66 ± 10	213/97	13
Kosuge. M et al. (2006) [34]	4	Japan	Cross-sectional	90 ST-elevation AVR ≥ 0.5 mm 243 non-ST-elevation AVR	67 ± 10	230/103	16
Kosuge. M et al. (2011) [31]	Unclear	Japan	Cross-sectional	157 ST-elevation AVR ≥ 0.5 mm 415 non-ST-elevation AVR	67 ± 11	397/115	15
Kosuge. M et al. (2001) [36]	5	Japan	Cross-sectional	23 ST-elevation AVR ≥ 0.5 mm 82 non-ST-elevation AVR	58	91/14	13
Ozmen.N et al. (2010)^*^ [40]	Unclear	Turkey	Case–control	21 ST-elevation AVR ≥ 0.5 mm 40 non-ST-elevation AVR	47.86	42/19	19
Nough. H et al. (2012) [39]	1	Iran	Cross-sectional	81 0.05 ≤ ST-elevation AVR < 1 mm 43 ST-elevation AVR ≥ 1 mm 276 non-ST-elevation AVR	61.0 ± 12	257/143	13
N. Misumida et al. (2016) [5]	1	Israel	Cross-sectional	97 ST-elevation AVR ≥ 0.5 mm 282 non-ST-elevation AVR	64.7	226/153	15
Rostoff. P et al. (2005) [43]	Unclear	Poland	Cross-sectional	54 ST-elevation AVR ≥ 0.5 mm 80 non-ST-elevation AVR	60.6 ± 9.5	92/42	12
Ruiz-Mateos. B et al. (2020) [44]	Unclear	Spain	Cohort	20 ST-elevation AVR 322 non-ST-elevation AVR	60	275/67	11
Separham. A et al. (2018) [45]	1	Iran	Cohort	122 ST-elevation AVR ≥ 0.5 mm 278 non-ST-elevation AVR	63.93 ± 13.05	285/115	14
Jalal. U. M et al. (2019) [47]	1	Bangladesh	Cross-sectional	6 ST-elevation AVR ≥ 0.5 mm 101 non-ST-elevation AVR	56.36	95/12	10
Wong. C et al. (2010) [48]	Unclear	New Zealand	Cohort	1685 ST-elevation AVR = 0.5 mm 819 ST-elevation AVR = 1 mm 325 ST-elevation AVR ≥ 1.5 mm 12,996 non-ST-elevation AVR	60.77	11,375/4450	15
Tang, et al. (2008) [46]	4.5	China	Cross-sectional	26 ST-elevation AVR ≥ 0.5 mm 30 non-ST-elevation AVR	65.92	33/17	14
Wong. C et al. (2012) [49]	Unclear	New Zealand	Cohort	1109 ST-elevation AVR ≥ 1 mm 14,206 non-ST-elevation AVR	60.77	Total = 15,315	13
Wu et al. (2008) [50]	Unclear	China	Cross-sectional	68 0.05 ≤ ST-elevation AVR < 1 mm 77 ST-elevation AVR ≥ 1 mm 281 non-ST-elevation AVR	58.92	292/134	14
Yamaji. H et al. (2002) [51]	Unclear	Japan	Cross-sectional	36 ST-elevation AVR ≥ 0.5 mm 12 non-ST-elevation AVR	–	Total = 48	13
Yan. T.A et al. (2007) [52]	5	13 countries	Cross-sectional	292 0.05 ≤ ST-elevation AVR < 1 mm 76 ST-elevation AVR ≥ 1 mm 4696 non-ST-elevation AVR	66.23	3199/1865	14
Zhang. X et al. (2015) [54]	4	China	Cross-sectional	192 ST-elevation AVR ≥ 0.5 mm 254 non-ST-elevation AVR	62.1 ± 12	335/91	14
Yan. Y et al. (2015) [53]	3	China	Cross-sectional	54 ST-elevation AVR ≥ 0.5 mm 141 non-ST-elevation AVR	62.96	112/83	13
Gaffari. S et al. (2016) [24]	1	Iran	Cohort	64 ST-elevation AVR < 1 mm 106 1 ≤ ST-elevation AVR < 2 mm 60 ST-elevation AVR ≥ 2 mm	55.96 ± 8.91	159/71	13
Alherbish. A et al. (2013) [12]	2	Canada, New Zealand, NC	Cohort	3819 non-ST-elevation AVR 352 ST-elevation AVR ≥ 1 mm	60.79	4289/1349	16
Cerit. L et al. (2017) [19]	3	Cyprus	Cross-sectional	37 non-ST-elevation AVR 80 ST-elevation AVR ≥ 0.5 mm	61.35	89/28	15
Barrabés. J et al. (2003) [17]	15	Spain	Cross-sectional	525 non-ST-elevation AVR 116 0.5 ≤ ST-elevation AVR < 1 mm 134 ST-elevation AVR ≥ 1 mm	61.3	592/183	14
Gachchhadar. P et al. (2018) [23]	1	Bangladesh	Cross-sectional	11 ST-elevation AVR < 0.75 mm 25 ST-elevation AVR > 0.75 mm	56.4	30/6	14
Goto. Y et al. (2011) [25]	6	Japan	Cross-sectional	106 non-ST-elevation AVR 85 ST-elevation AVR ≥ 0.5 mm	62 ± 10	185/52	14
Hengrussamee A et al. (2005) [26]	1	Thailand	Cross-sectional	17 non-ST-elevation AVR 9 ST-elevation AVR ≥ 0.5 mm	64 ± 9	21/5	11
Baheti. A et al. (2021) [16]	2	America	Cohort	567 non-ST-elevation AVR 74 ST-elevation AVR ≥ 1 mm	57	366/275	15
Ducas. R et al. (2013) [22]	4	Canada	Cohort	138 non-ST-elevation AVR 53 ST-elevation AVR ≥ 0.5 mm	68	132/59	14
Hirano. T et al. (2006) [27]	16	Japan	Cross-sectional	101 non-ST-elevation AVR 39 ST-elevation AVR ≥ 0.5 mm	65	96/44	13
Chen. Y et al. (2004) [21]	15	Taiwan	Cross-sectional	6 non-ST-elevation AVR 16 ST-elevation AVR > 0.5 mm	63.68	18/4	12
Badri. M et al. (2019) [15]	2	Egypt	Cross-sectional	21 non-ST-elevation AVR 39 ST-elevation AVR ≥ 0.5 mm	60 ± 9	37/28	15
Aygül. N et al. (2006) [13]	4	Turkey	Cross-sectional	315 non-ST-elevation AVR 46 ST-elevation AVR ≥ 0.5 mm	58 ± 10	293/68	13
Aygul. N et al. (2008) [14]	6	Turkey	Cross-sectional	795 non-ST-elevation AVR 155 ST-elevation AVR > 0.5 mm	59 ± 12	742/208	14
Sheibani. H et al. (2021) [28]	1	Iran	Cross-sectional	335 non-ST-elevation AVR 137 ST-elevation AVR ≥ 0.5 mm	61 ± 14	248/224	12
Beyranvand. M et al. (2017) [18]	3	Iran	Cross-sectional	116 non-ST-elevation AVR 79 0.5 ≤ ST-elevation AVR < 1 mm 24 ST-elevation AVR ≥ 1 mm	59.00 ± 13.14	228/60	12
Chen. Y et al. (2005) [20]	7	Taiwan	Cross-sectional	38 non-ST-elevation AVR 18 0.5 ≤ ST-elevation AVR < 1 mm 22 ST-elevation AVR ≥ 1 mm	63.68	18/4	10
Abbase. A et al. (2011) [11]	1	Iraq	Cross-sectional	56 None 43 ST-elevation AVR ≥ 0.5	56.44	65/35	12

### Main analysis

**Fig. 2:**
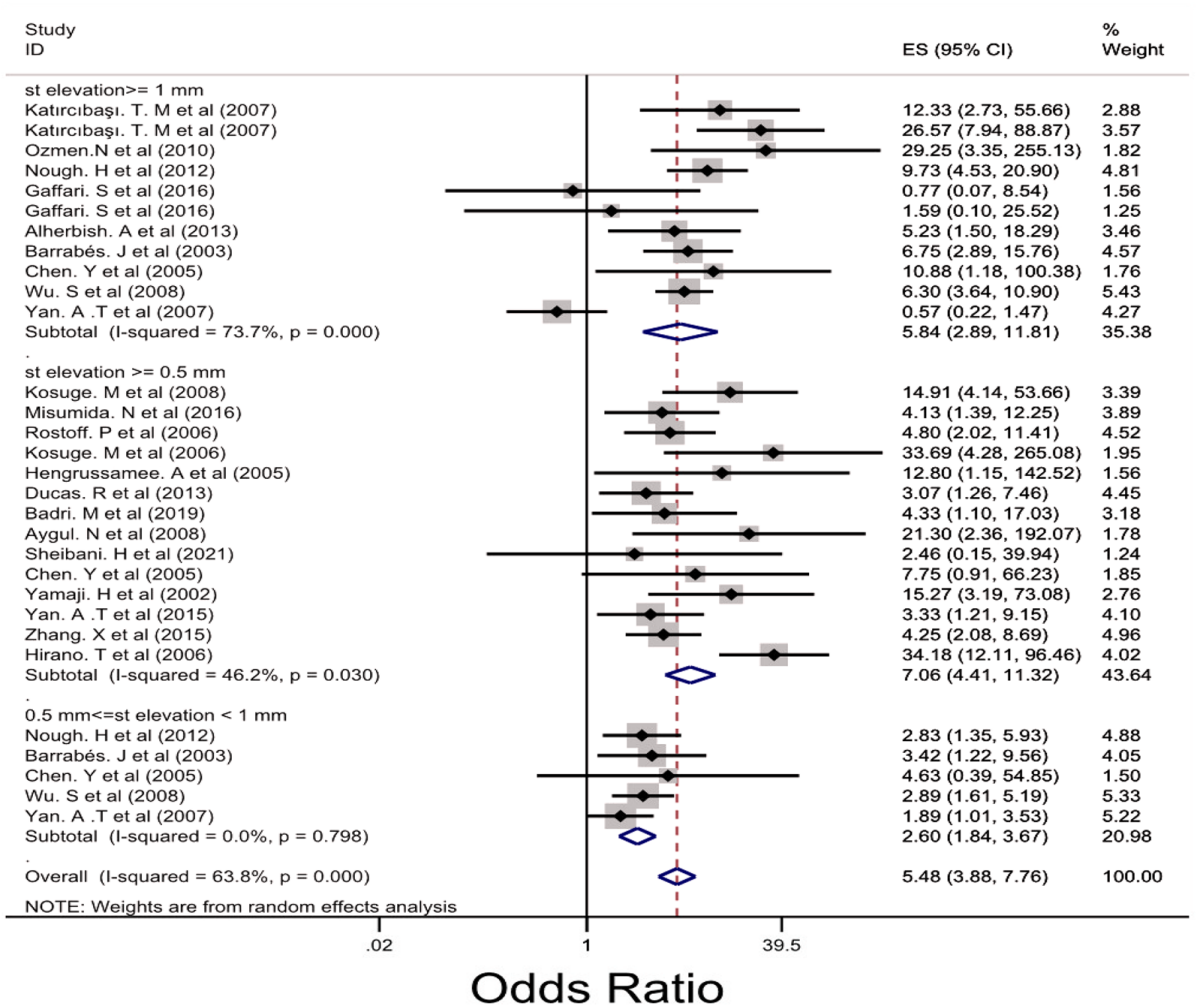
The forest plot represents the odds of left main disease in different size of STE in lead aVR

**Fig. 3:**
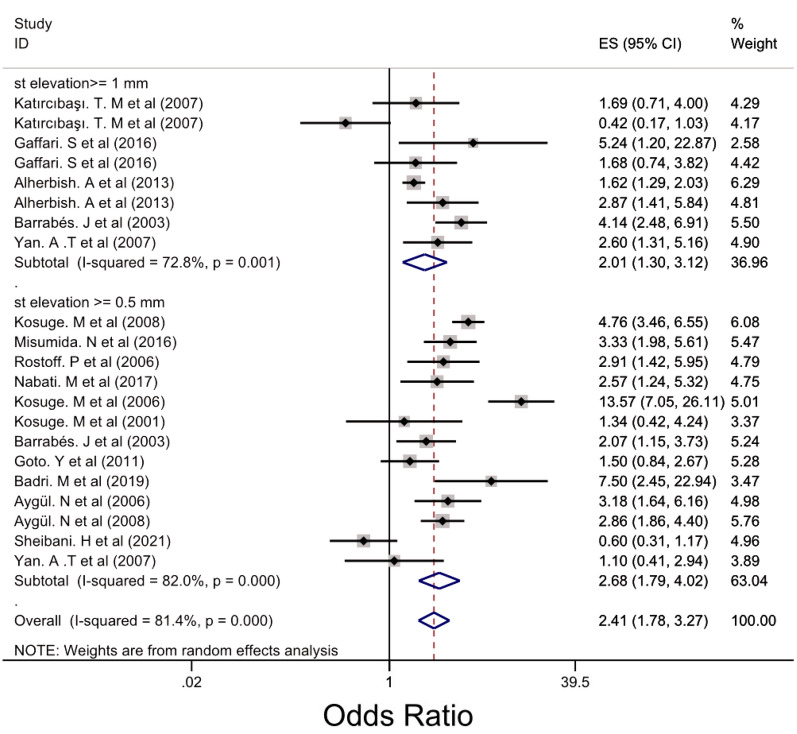
The forest plot reports the odds of three-vessel disease in variant subgroups of ST-elevation in lead aVR

**Fig. 4:**
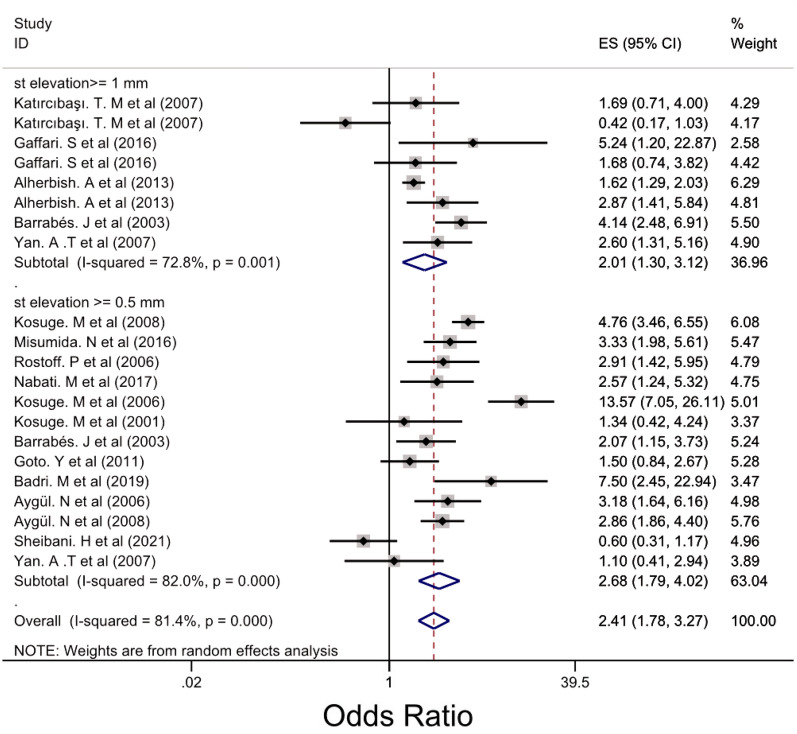
The forest plot of the association between LM/3VD and ST-elevation in lead aVR

**Fig. 5:**
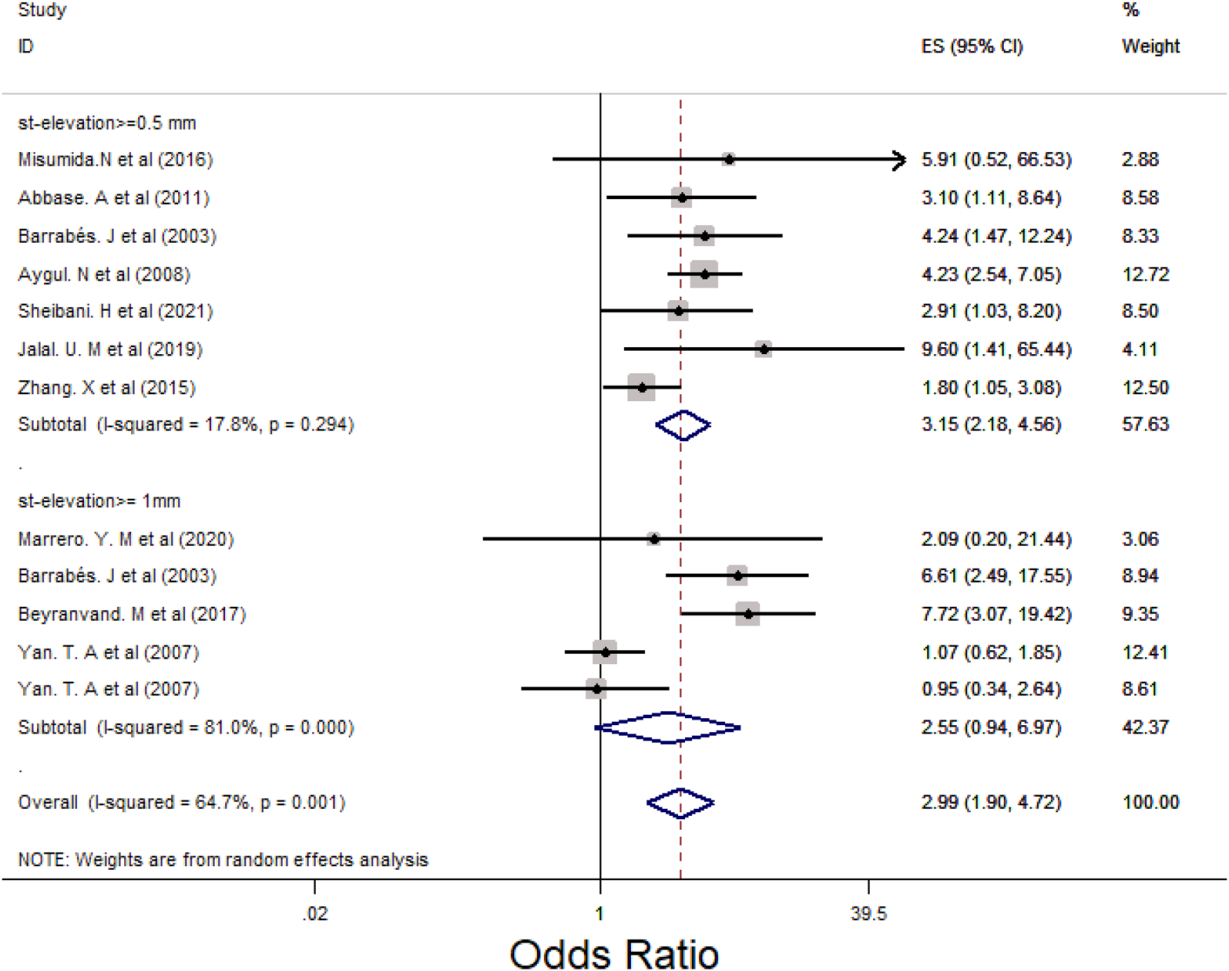
The forest plot of the association between in-hospital mortality and subgroups of ST-elevation in lead aVR

**Fig. 6:**
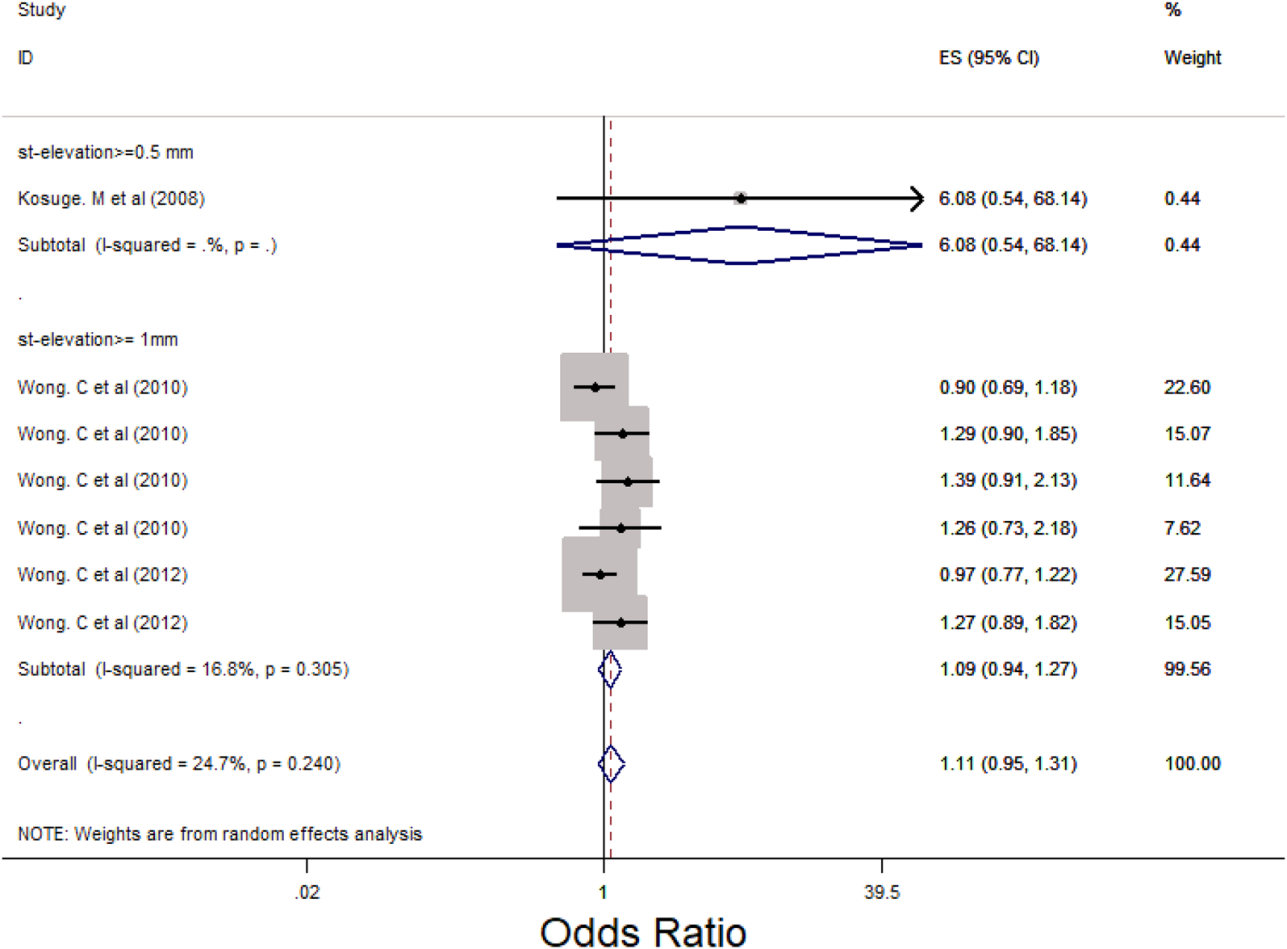
The forest plot represents the odds of 30-day mortality in different subgroups of ST-elevation in lead aVR

**Fig. 7:**
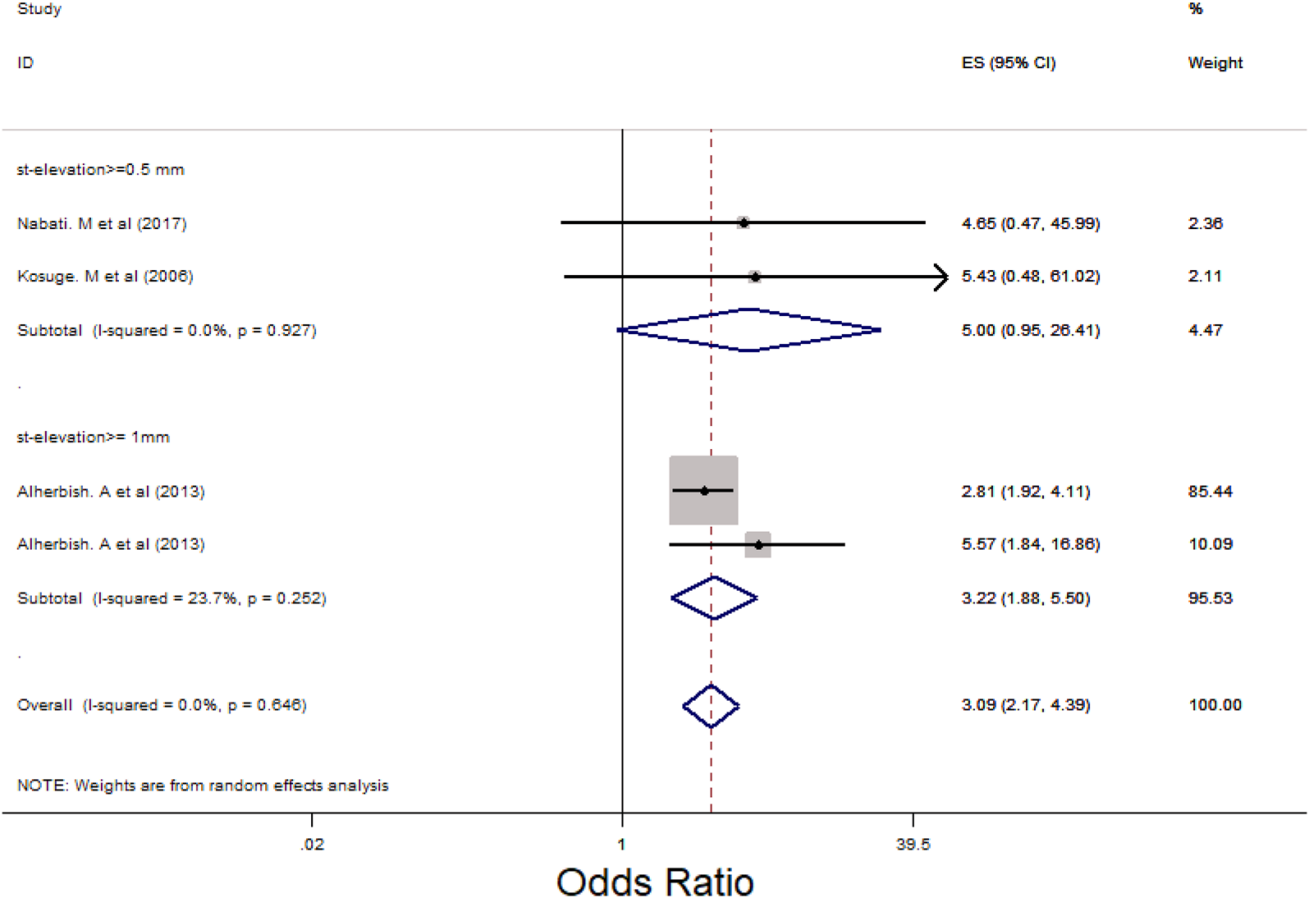
The forest plot reports the odds of 90-day mortality in different size of ST-elevation in lead aVR

Subgroup analysis was performed for LM, 3VD and LM/3VD based on the size of STE in lead aVR (Figs. 2, 3 and 4). For LM, the overall OR was 5.48 (95% CI 3.88, 7.76). STE > 0.5 mm groups had higher OR compared with STE > 1 mm and 0.5 < STE < 1 mm (Fig. 2) and the heterogenicity between the studies was significant (*I*^2^ = 63.8%, *p* = 0.000). For 3VD, the overall OR was 2.41 (95% CI 1.78, 3.27) (Fig. 3) and the overall heterogenicity was significant between the studies (*I*^2^ = 81.4%, *p* = 0.0000). Also, STE > 0.5 mm had higher OR than STE > 1 mm. And finally for LM/3VD, with a significant heterogenicity (*I*^2^ = 86.3%, *p* = 0.0000), the overall OR was 6.21 (95% CI 3.49, 11,6). Similar to LM and 3VD, STE > 0.5 mm had higher OR compared with the other subgroup (Fig. 4). From the all 45 studies, 15 studies (with 30,521 participants) reported death. Despite STE in lead aVR could not predict 30-day mortality (OR = 1.11, CI 0.95, 1.31, *I*^2^ = 24.7%), STE in lead aVR was meaningfully associated with in-hospital death (OR = 2.99, CI 1.90, 4.72, *I*^2^ = 64.7%) and 90-day mortality (OR = 3.09, CI 2.17, 4.39, *I*^2^ = 0.0%) (Figs. 5, 6 and 7).

### Sensitivity and specificity

The overall sensitivity of STE for LM was 0.77 (95% CI 0.65, 0.89, *I*^2^ = 63.8%). The cut-off points of 1 mm had the highest sensitivity (0.9, 95% CI 0.82, 0.98) and negative predictive value (NPV) (0.94, CI 0.89, 0.99) (Additional file 1: Figures S5, S8). Pooled specificity for LM was 0.71 (95% CI 0.61, 0.81, I^2 ÷^ = 97%) with the highest specificity for 0.5 mm cut-off points (0.75, 95% CI 0.67, 0.84, *I*^2^ = 97%) (Additional file 1: Figure S6). The positive predictive value (PPV) of STE in lead aVR for LM was 0.5 (95% CI 0.36, 0.63, *I*^2^ = 97.4%) (Additional file 1: Figure S7). Pooled sensitivity for LM/3VD was 0.52 (95% CI 0.35, 0.68, *I*^2^ = 99.5%) and the cut-off points of 0.5 mm had the highest sensitivity (0.76, 95% CI 0.61, 0.90) and NPV (0.89, 95% CI 0.83, 0.94) (Additional file 1: Figures S1, S4). The NPV of STE in lead aVR for LM/3VD was 0.86 (95% CI 0.82, 0.90, *I*^2^ = 97%). Also, the cut-off points of 1.5 mm had the highest specificity (0.80, 95% CI 0.75, 0.85) and PPV (0.57, 95% CI 0.42, 0.71) (Additional file 1: Figures S2, S3).

To investigate the possible causes of heterogeneity, meta-regression analysis was performed based on sex, country, total sample size, mean age and publish year. There was not any significant source of heterogenicity except for total sample size in LM odds ratio (*p* = 0.011), LM/3VD odds ratio (*p* = 0.002) and LM npv (*p* = 0.045). Additionally, publish year was also meaningful for LM/3VD odds ratio (*p* = 0.007).

### Publication bias

Publication bias was evaluated by the Begg's test and funnel‐plot interpretation. No significant bias was found among those articles with LM (*p* = 0.31), 3VD (*p* = 0.29) and LM/3VD (*p* = 0.26) in Begg’s test. Furthermore, there was no meaningful bias with regard to in-hospital mortality (*p* = 0.13), 30-day mortality (*p* = 0.18), and 90-day mortality (*p* = 0.94).

## Discussion

Fast diagnosis of cardiovascular disease plays a critical role in rescuing of patients, especially patients with coronary artery disease [28]. aVR lead that is often ignored in clinic, pose some key date [55].

Our finding demonstrated STE in lead aVR can predict LM and LM/3VD with higher odds ratio than 3VD (OR_LM_: 5.48, CI [3.88, 7.76], OR_LM/EVD_ = 6.21, CI [3.49, 11.06], OR_3VD_ = 2.41, CI [1.78, 3.27]). Also, Misumida N et al. declared STE in lead aVR as an independent diagnosis factor of LM/3VD in non-STEMI (OR = 2.99, CI [1.79, 4.98]). In addition, Rathi N et al. from Pakistan represented that the number of LM/3VD patients in STE-aVR group was significantly higher than non-STE aVR group (51 (62.96%) vs 9 (29.03%), *p* < 0.0001) [41]. In a single cohort study from Mazandaran province, there was not any meaningful difference in both groups (STE-aVR vs non-STE aVR) in terms of LM/3VD. By the way, they pointed that STE in lead aVR related to severity of atherosclerosis, however their sample size was small [38]. Besides, another study with larger sample size (*n* = 472) could not found significant relationship between angiography result and STE in lead aVR [28]. Moreover, a systematic review and meta-analysis recently demonstrated that not only STE in AVR is related to LM but also the degree of elevation is effective, which is consistent with our results (OR _STE> 1 mm_ = 4.17, CI [3.04, 5.70], OR _0.5 mm<STE<1 mm_ = 2.57, CI [1.97, 3.36] [56].

Misumida N et al. showed that STE in lead aVR could not make any meaningful change in the number of in-hospital mortality (*p* = 0.16) [5]. Moreover, another study with a large sample size (*n* = 15,315) reported that there was not any significant relationship between STE in lead aVR and 30-day mortality in adjusted model [49]. By contrast, a Spanish study pointed increase in the number of death related to STE in lead aVR significantly (*p* = 0.04). Also, another retrospective cross-sectional study represented that the chance of mortality in patients with STE upper than 1 mm was 7. 72 (CI [ 3.07, 19.42, *P* < 001) [18]. Besides, one study from New Zealand declared that mortality rate in inferior myocardial infarction was along with STE in lead aVR in adjusted model (hazard ratio = 5.87, CI [2.09–16.5]) [12]. In our study, the chance of in-hospital and 90-day mortality increased unlike 30-day mortality (OR_in-hospital_ = 2.99, CI [1.90, 4.72], OR_90-day_ = 3.09, CI [2.17, 4.39]).

Katırcıbaşı T. M et al. represented sensitivity and for diagnosis of LM disease in patients with 0.5 mm STE in lead aVR were 92.9% and 48.6%, respectively [30]. One study from Iran had similar results for detection of LM (sensitivity = 100, specificity = 33.5%) [24]. However, their cut-off point was 1 mm in order to consider STE in lead aVR, that was upper than prior study (cut-off = 0.5 mm). Besides, another study considered 0.5 mm elevation as a significant STE and had similar sensitivity (80%) and higher specificity (92.3%) [27]. Hussien A et al. declared that sensitivity and specificity were 77% and 65% when the cut-off point was considered 0.5 mm for detecting LM/3VD. Also, when they set higher cut-off point (> 1.5 mm), sensitivity and specificity reach to 14% and 98%, respectively. Likewise, cut-off point of 0.5 mm and 1.5 mm had highest NPV and PPV, respectively (78%, 91%) [29]. Kosuge M et al. followed the same pattern. Thus, 0.5 mm STE had highest sensitivity and NPV (91%, 99%, respectively) and 1.5 mm STE had highest specificity and PPV (98%, 58%, respectively) for diagnosis of LM/3VD. [31]. In this regard, the results of Misumida N et al.’s study were concordant with previous studies in this regard. A systematic review and meta-analysis showed overall sensitivity of LM and LM/3VD was 39% and 40%, respectively. Moreover, the overall specificity of LM and LM/3VD was 86% and 82%, respectively [57]. Our results represented overall sensitivity of LM and LM/3VD was 77% and 52%, respectively. And also, the overall specificity of LM/3VD was higher than LM (89% Vs 71%). Furthermore, STE ≥ 0.5 mm and STE ≥ 1 mm had the highest sensitivity for LM/3VD and LM (sensitivity _LM_ = 90%, sensitivity _LM/3VD_ = 76%). Additionally, cut-off points of 1 mm STE in lead aVR had the highest NPV (94%) and PPV (53%) with regard to LM. However, cut-off points of 0.5 mm and 1 mm STE in lead aVR had the highest NPV (89%) and PPV (75%) in terms of LM/3VD, respectively.

### Limitation

In this study, we were not able to access the full text of some studies that might change our result.

## Conclusion

STE in lead aVR increases the risk of LM and LM/3VD more than 3VD. Furthermore, STE ≥ 0‌0.5 mm and STE ≥ 1 mm were the best cut-off points to screen patients in terms of LM/3VD and LM, respectively. Additionally, the overall specificity of LM/3VD was greater than LM.

## Supplementary Information

**Additional file 1: Figure S1. **Forest plot represents pooled sensitivity of LM/3VD in different subgroups of ST-elevation in lead aVR. Left main/three vessel disease: LM/3VD. **Figure S2.** Forest plot is showing the pooled specificity of LM/3VD in variant size of ST-elevation in lead aVR. Left main/three vessel disease: LM/3VD. **Figure S3.** The forest plot is showing the pooled positive predictive value of LM/3VD according to the size of ST-elevation in lead aVR. Left main/three vessel disease: LM/3VD. **Figure S4.** Forest plot is showing the pooled negative predictive value of LM/3VD according to ST-elevation in lead aVR subgroups. Left main/three vessel disease: LM/3VD. **Figure S5.** Forest plot is showing the pooled sensitivity of LM according to ST-elevation in lead aVR subgroups. **Figure S6.** The forest plot is showing the pooled specificity of LM according to the ST-elevation in lead aVR subgroups. **Figure S7.** The forest plot is showing the pooled positive predictive value of LM according to the size of ST-elevation in lead aVR. Left main: LM. **Figure S8.** Forest plot is showing the pooled negative predictive value of LM according to ST-elevation in lead aVR subgroups. Left main: LM.

## Data Availability

All data generated or analyzed during this study are included in this published article [and its supplementary information files].

## Abbreviations

STE: St-elevation
LM: Left main
3VD: Three-vessel disease
ACS: Acute coronary syndrome
NPV: Negative predictive value
PPV: Positive predictive value
OR: Odds ratio
CI: Confidence interval
IHD: Ischemic heart disease
NSTEMI: Non-ST elevation myocardial infarction
STEMI: ST-elevation myocardial infarction
LMCA: Left main coronary artery stenosis
Electrocardiogram: ECG
LAD: Left anterior descending
