# Supplementary material for: The prognostic effect of ST-elevation in lead aVR on coronary artery disease, and outcome in acute coronary syndrome patients: a systematic review and meta-analysis
Source: Eur J Med Res. 2022 Dec 21;27:302. doi: 10.1186/s40001-022-00931-5 (PMC9769006; doi:10.1186/s40001-022-00931-5)
Supplement: Supplementary file 1 — Additional file 1: Figure S1. Forest plot represents pooled sensitivity of LM/3VD in different subgroups of ST-elevation in lead aVR. Left main/three vessel disease: LM/3VD. Figure S2. Forest plot is showing the pooled specificity of LM/3VD in variant size of ST-elevation in lead aVR. Left main/three vessel disease: LM/3VD. Figure S3. The forest plot is showing the pooled positive predictive value of LM/3VD according to the size of ST-elevation in lead aVR. Left main/three vessel disease: LM/3VD. Figure S4. Forest plot is showing the pooled negative predictive value of LM/3VD according to ST-elevation in lead aVR subgroups. Left main/three vessel disease: LM/3VD. Figure S5. Forest plot is showing the pooled sensitivity of LM according to ST-elevation in lead aVR subgroups. Figure S6. The forest plot is showing the pooled specificity of LM according to the ST-elevation in lead aVR subgroups. Figure S7. The forest plot is showing the pooled positive predictive value of LM according to the size of ST-elevation in lead aVR. Left main: LM. Figure S8. Forest plot is showing the pooled negative predictive value of LM according to ST-elevation in lead aVR subgroups. Left main: LM. [file 40001_2022_931_MOESM1_ESM.docx]

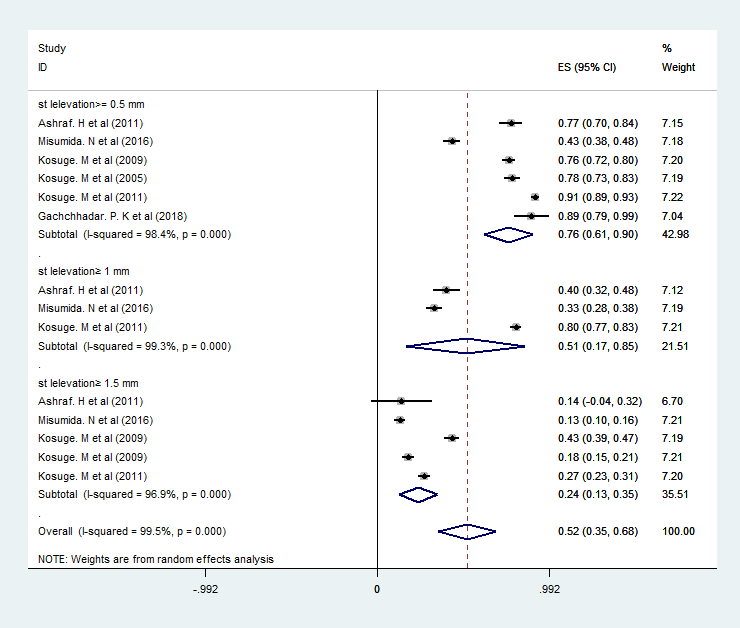


Supplementary fig 1: forest plot represents pooled sensitivity of LM/3VD in different subgroups of ST-elevation in lead aVR. Left main/three vessel disease:LM/3VD.


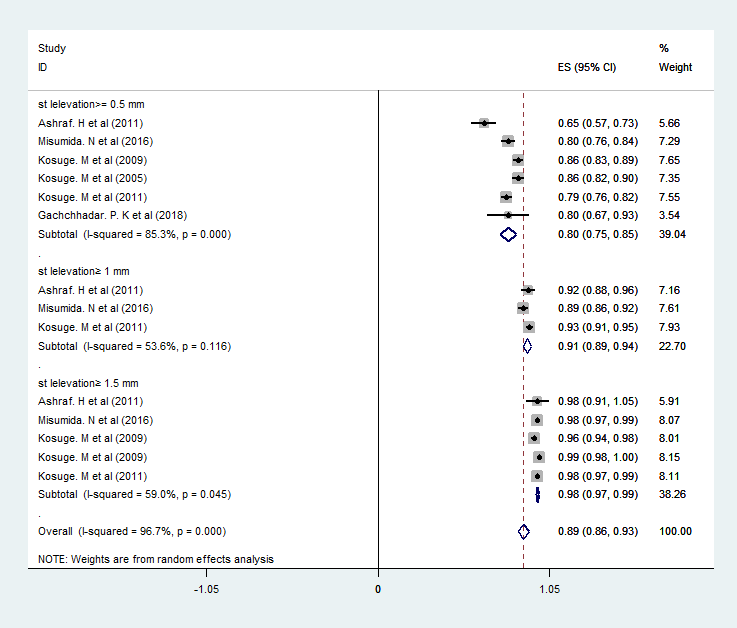


Supplementary fig 2: forest plot is showing the pooled specificity of LM/3VD in variant size of ST-elevation in lead aVR. Left main/three vessel disease:LM/3VD.


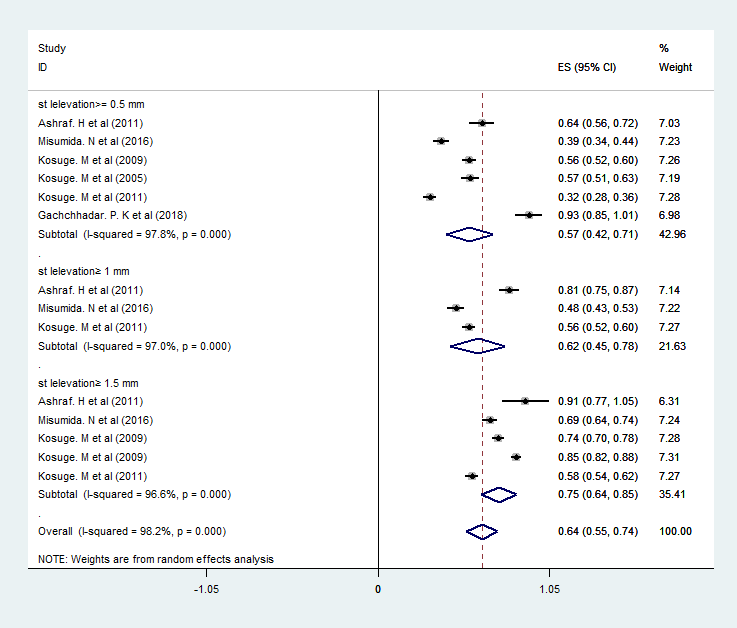


Supplementary fig 3: the forest plot is showing the pooled positive predictive value of LM/3VD according to the size of ST-elevation in lead aVR. Left main/three vessel disease:LM/3VD.


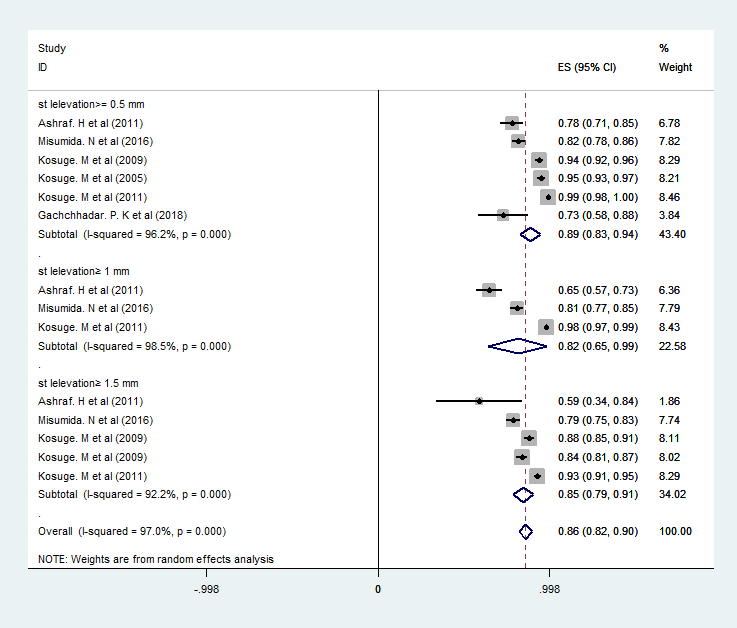


Supplementary fig 4: forest plot is showing the pooled negative predictive value of LM/3VD according to ST-elevation in lead aVR subgroups. Left main/three vessel disease:LM/3VD.


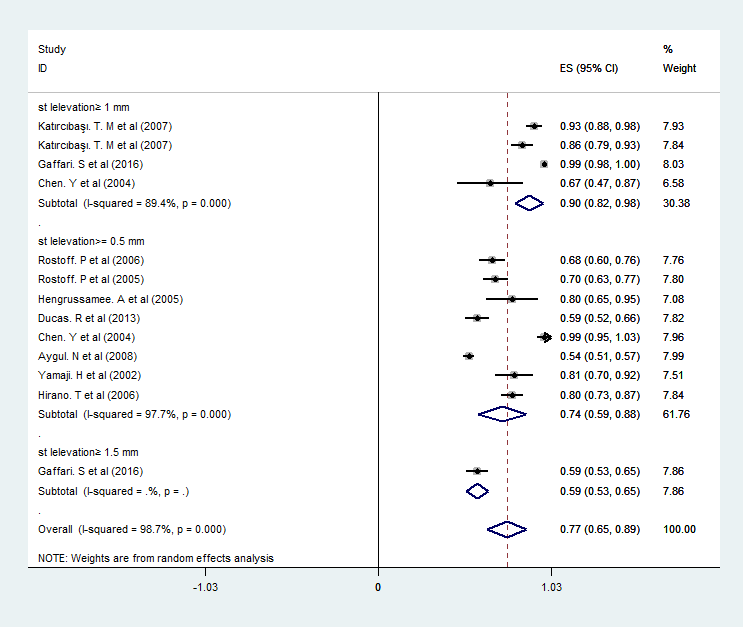


Supplementary fig 5: forest plot is showing the pooled sensitivity of LM according to ST-elevation in lead aVR subgroups.


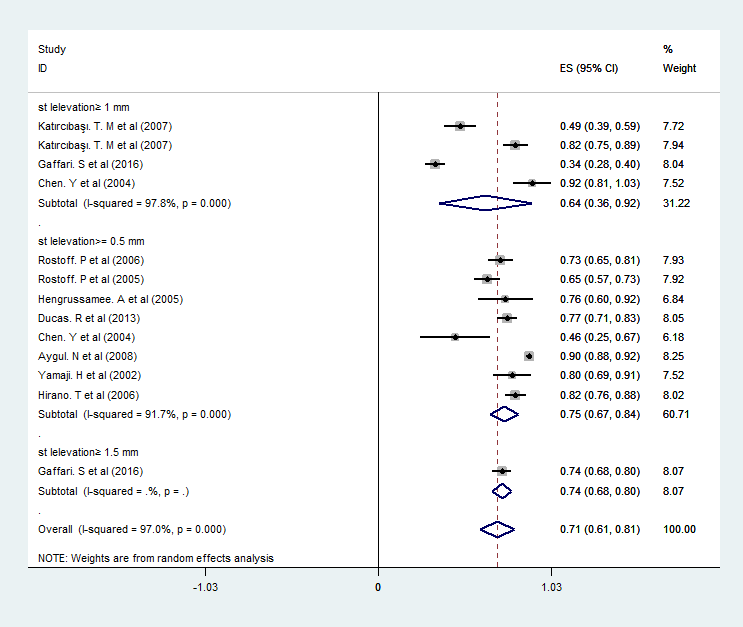


Supplementary fig 6: the forest plot is showing the pooled specificity of LM according to the ST-elevation in lead aVR subgroups.


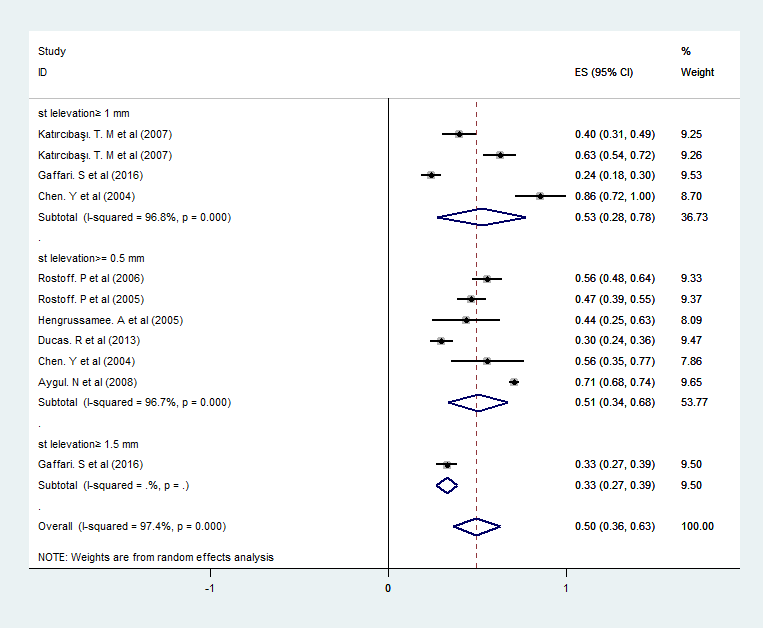


Supplementary fig 7: the forest plot is showing the pooled positive predictive value of LM according to the size of ST-elevation in lead aVR. Left main:LM


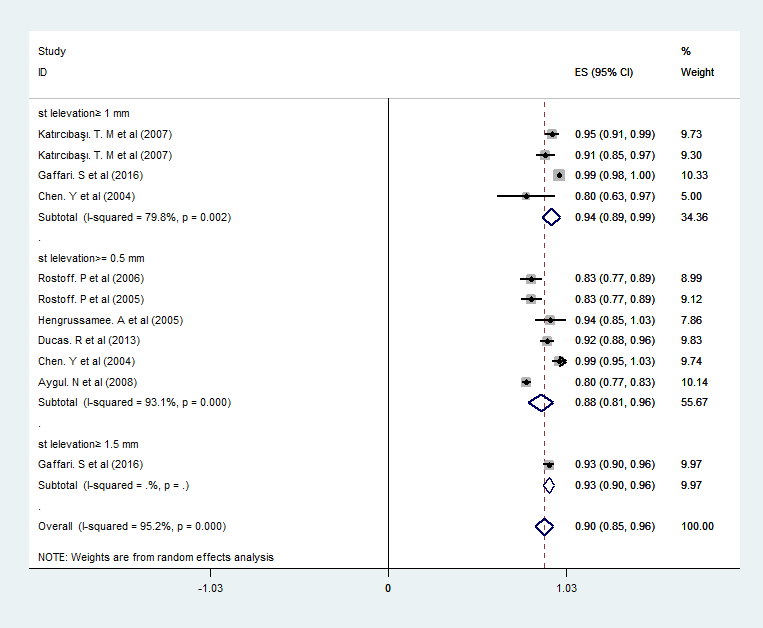


Supplementary fig 8: forest plot is showing the pooled negative predictive value of LM according to ST-elevation in lead aVR subgroups. Left main:LM
